# Mammalian Neuraminidases in Immune-Mediated Diseases: Mucins and Beyond

**DOI:** 10.3389/fimmu.2022.883079

**Published:** 2022-04-11

**Authors:** Erik P. Lillehoj, Irina G. Luzina, Sergei P. Atamas

**Affiliations:** ^1^ Department of Pediatrics, University of Maryland School of Medicine, Baltimore, MD, United States; ^2^ Department of Medicine, University of Maryland School of Medicine, Baltimore, MD, United States; ^3^ Research Service, Baltimore Veterans Affairs (VA) Medical Center, Baltimore, MD, United States

**Keywords:** post-translational modification, sialidase, sialic acid, fibrosis, inflammation, infection, autoimmunity

## Abstract

Mammalian neuraminidases (NEUs), also known as sialidases, are enzymes that cleave off the terminal neuraminic, or sialic, acid resides from the carbohydrate moieties of glycolipids and glycoproteins. A rapidly growing body of literature indicates that in addition to their metabolic functions, NEUs also regulate the activity of their glycoprotein targets. The simple post-translational modification of NEU protein targets—removal of the highly electronegative sialic acid—affects protein folding, alters protein interactions with their ligands, and exposes or covers proteolytic sites. Through such effects, NEUs regulate the downstream processes in which their glycoprotein targets participate. A major target of desialylation by NEUs are mucins (MUCs), and such post-translational modification contributes to regulation of disease processes. In this review, we focus on the regulatory roles of NEU-modified MUCs as coordinators of disease pathogenesis in fibrotic, inflammatory, infectious, and autoimmune diseases. Special attention is placed on the most abundant and best studied NEU1, and its recently discovered important target, mucin-1 (MUC1). The role of the NEU1 - MUC1 axis in disease pathogenesis is discussed, along with regulatory contributions from other MUCs and other pathophysiologically important NEU targets.

## Introduction

**Figure 1:**
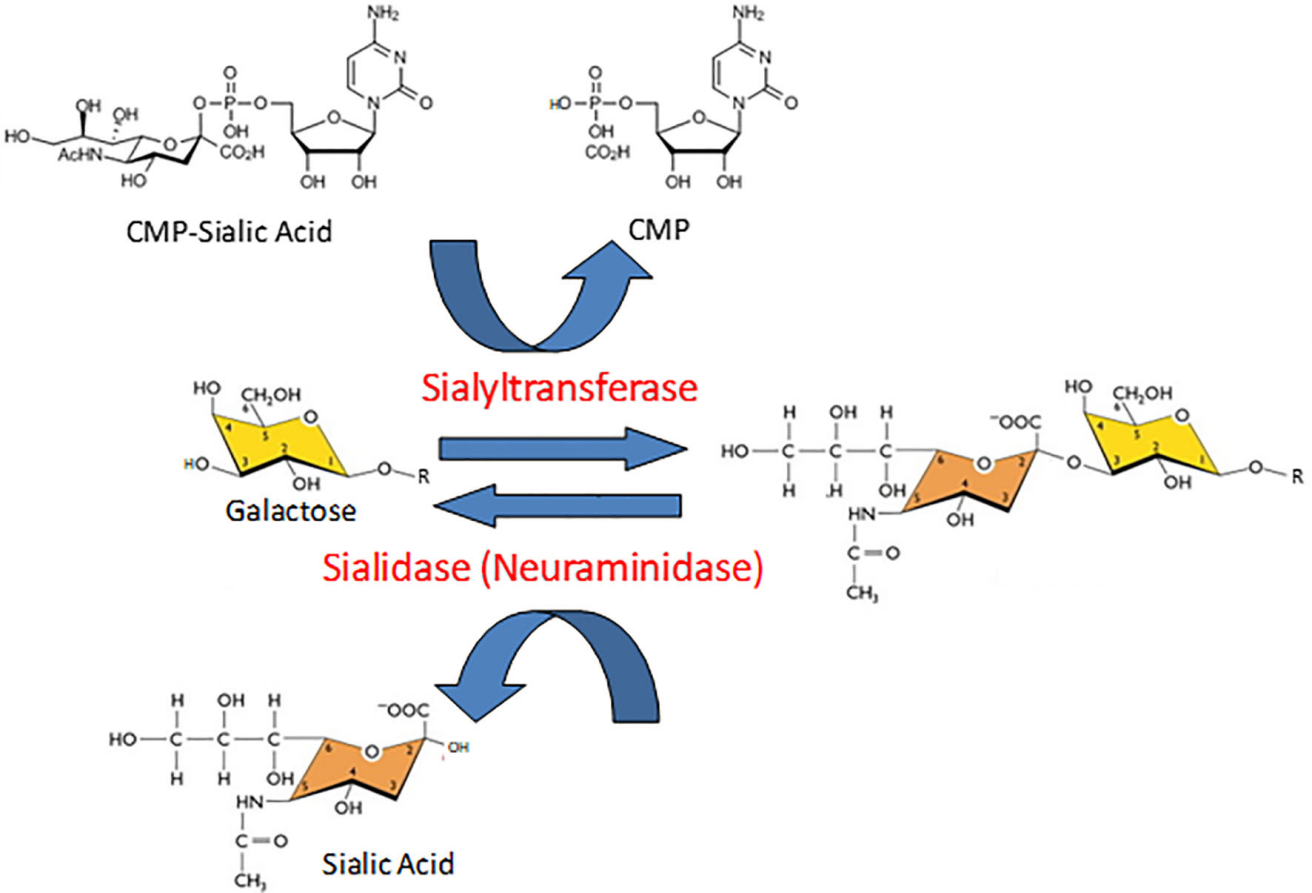
Opposing catalytic activities of sialyltransferases and sialidases. In the reaction catalyzed by sialyltransferases, sialic acid is transferred from its activated nucleotide sugar donor, cytidine 5’-monophosphate (CMP)-sialic acid, to the penultimate galactose (illustrated here) or N-acetylgalactosamine residue of a glycan chain, thereby releasing free CMP. The resulting sialic acid-galactose disaccharide is illustrated in an α(2-3) linkage. In the sialidase-catalyzed reaction, the sialic acid-galactose/N-acetylgalactosamine covalent bond is hydrolyzed to released release free sialic acid.

Protein glycosylation is a biologically ubiquitous, enzymatic post-translational modification involving the attachment of one or more carbohydrate moieties to a protein molecule, typically at the nitrogen atom of the side chain amide group of an Asn residue (N-linked glycosylation) or the oxygen atom of the side chain hydroxyl group of a Ser or Thr residue (O-linked glycosylation). Glycosyltransferases catalyze the transfer of the glycan to the protein acceptor, while glycosidases catalyze the reverse reaction. Sialyltransferases and neuramindases (NEUs), also known as sialidases, are subclasses of glycosyltransferases and glycosidases, respectively, that specifically add or remove sialic acids to/from the terminal position of glycan chains (**Figure 1**). Sialic acids comprise a family of more than 50 closely related, but structurally diverse, nine-carbon monosaccharides (1–3). N-acetylneuraminic acid (Neu5Ac or NANA) is the predominant sialic acid in most mammalian cells and is often used interchangeably with sialic acid. Most commonly, the C-2 carbon atom of the α-anomer of Neu5Ac forms a covalent bond with the C-3 hydroxyl group of galactose (Gal), or the C-6 hydroxyl group of Gal or N-acetylgalactosamine (GalNAc), to form α(2,3)- and α(2,6)-linked structures. In a small number of proteins, polysialic acid has been identified as a unique, α(2,8)-linked homopolymer of up to 150 sialic acid residues that confers an exceptionally high electronegative charge (4). With their terminal location and negative charge at physiological pH, sialic acids are strategically positioned to influence intermolecular and intercellular interactions through steric hindrance and/or electronic effects. Consequently, sialic acids, and the enzymes that add or remove them from glycan chains, influence protein tertiary conformation, bioactivity, and proteolysis.

## NEUs – General Features

As early as 1960, mammalian sialidase activity was detected in fractioned human serum (5). Later, crude sialidase activity was reported in a variety of eukaryotic cell and tissue extracts (6–11). Prior to isolation and characterization of mammalian *NEU* genes, the biochemical characterization and enrichment/purification of these various sialidases demonstrated the presence of enzymes with different molecular weights, subcellular localization, substrate specificities, and pH optimums *in vitro* (12). Klotho, a protein originally associated with senescence and aging, also has been reported to exhibit sialidase activity, although it lacks conserved structural domains characteristic of canonical NEU proteins (13).

NEUs are found in organisms of diverse evolutionary lineages, from viruses to mammals, and functional consequences of their enzymatic activity across and within taxa are remarkably broad and complex. There are four mammalian NEUs, NEU1 – NEU4, and they are all exosialidases, meaning that they remove the terminal sialic residues from the glycan moieties of glycoproteins and glycolipids. Mammalian NEUs are differentially expressed across tissues and differentially distributed at the subcellular level. Their relative expression levels are usually different, with NEU1 being the predominant isoform, often closely followed by the relatively abundant NEU3, whereas NEU2 and NEU4 are commonly expressed minimally if at all; this generalization is not always supported as it may be hindered by specifics of a particular disease process, tissue involved, or differentiation state of the cells engaged. Based on their catalytic function, NEUs are primarily metabolic enzymes; mutations of the NEU1 gene lead to a metabolic disorder, sialidosis (14). The research of the past two decades has revealed that NEUs also regulate important biological functions and are involved in disease pathogenesis (12, 15–17). This is not surprising, considering that many homeostatic mediators involved in metabolic regulation, development, reproduction, and immune tolerance, as well as pathophysiological regulators involved in immune and inflammatory diseases, fibrosis, atherosclerosis, cancer, and other diseases are sialylated glycoproteins. NEU targets and binding partners include multiple glycoproteins known to play important roles in the mechanisms of immunity, inflammation and fibrosis (**Table 1**).

**Table 1:** Selected confirmed and putative glycoprotein substrates of NEUs and NEU interactors with regulatory contributions to immune, inflammatory, and fibrotic processes.

Glycoprotein	NEU Isozyme	References
ApoB100	NEU1, NEU3	(18)
ATG5	NEU2	(19)
CD5	NEU1	(20)
CD18 (ITGB2)	NEU1, NEU3	(21–24)
CD31 (PECAM1)	NEU1	(22, 25)
CD36	NEU1	(22)
CD42b (GPIbα)	NEU1, NEU3	(26, 27)
CD44	NEU1	(28–30)
CD54 (ICAM1)	NEU1	(23)
CD64 (FCγR)	NEU1	(31)
CD104 (ITGB4)	NEU1	(32)
CD107a/b (LAMP-1, LAMP-2)	NEU1	(33, 34)
CD140 (PDGFR)	NEU1	(35)
CD220 (insulin receptor)	NEU1	(36–39)
CD221 (IGF-1R)	NEU1	(35, 36)
EGFR	NEU1, NEU3	(40–42)
HGFR/Met	NEU1	(43)
MMP9	NEU1	(22, 41, 44–47)
MUC1	NEU1	(40, 48–51)
TGF-β/LAP	NEU3	(52)
TLR2	NEU1	(22, 53)
TLR3	NEU1	(53)
TLR4	NEU1	(45, 47, 53–58)
TLR7	NEU1	(44)
TLR9	NEU1	(44)
TrkA	NEU1	(46, 59)

At the mechanistic level, the downstream effects of desialylation are substantially defined by the properties of sialic acid, which is a highly electronegative sugar. Desialylation affects the charge of the target molecules and even the often much sialylated cell surface. At the molecular level, desialylation can directly unmask or mask glycoproteins’ bindings sites for their molecular partners. Desialylation can also affect intramolecular interactions and, subsequently, protein folding, again revealing or sequestering binding sites for low molecular weight ligands or high molecular weight interactors, thus affecting the target glycoproteins’ functions as enzymes, receptors, or signal transducers. More specifically, several mechanistic pathways can be envisioned and have been experimentally validated (12, 15–17) through which neuraminidases control their glycoprotein targets and their downstream functioning. Desialylation also affects the target glycoproteins’ folding, thus additionally regulating protein-protein interactions. Desialylation of the entire cell surface has far-going consequences for cell-cell interactions, thus affecting pathophysiological processes on the large scale. Such changes in folding may induce transitions between functionally active and inactive states of enzymes, attenuation or augmentation of ligand binding by receptors, and masking or unmasking of proteolytic sites. On a more direct level, siglecs bind to sialic acid of glycoproteins, whereas galectins bind to Gal residues which become exposed following desialylation, as Gal is typically the subterminal carbohydrate to which the terminal sialic acid is attached in the glycan portions of glycoproteins and glycolipids. Thus, NEU-mediated desialylation diminishes siglec binding and allows for galectin binding to glycoproteins, affecting such interactions that are known to regulate immunity and inflammation.

Originally, the four mammalian NEUs were characterized by their characteristic subcellular localization, including lysosomal NEU1, cytosolic NEU2, plasma membrane NEU3, and lysosomal, mitochondrial, and endoplasmic reticular NEU4 (12). In the lysosome, NEU1 is associated with its chaperone/transport protein, protective protein/cathepsin A (PPCA), and β-galactosidase (60). It became quickly recognized that in addition to such predominant localization, each NEU may be distributed more broadly. For example, in addition to the predominantly membrane-targeted NEU3, the other NEUs can be found on the cell surface, including NEU1 (20, 41, 44–47, 53, 55, 56, 60–75), NEU2 (76–78), and NEU4 (79, 80). NEU1 on the plasma membrane has been reported either in the presence (62, 64–66, 72, 75) or absence (68, 71) of PPCA. Membrane localization allows NEUs to act as structural and functional modulators of extracellular soluble and membrane-bound molecules (17). Further information on the subcellular, cellular, and tissue distribution, substrate specificity, catalytic properties, and amino acid homologies of the four mammalian NEUs can be found in prior review articles (12, 15, 81–87).

## MUCs – General Features

Mucus is a complex mixture of ions, salts, peptides, proteins, glycoconjugates, and water covering the surface of mucosal tissues. The primary protein component of mucus are mucins, high molecular weight, extensively glycosylated proteins containing variable numbers of tandem repeats (VNTRs) in which Ser, Thr, and Pro amino acids are highly enriched. Mucin glycosylation primarily occurs through O-glycosidic linkages between the first GalNAc residue of the oligosaccharide side chains and Ser and Thr amino acids of the polypeptide backbone. More than 20 mucin glycoproteins have been identified and are broadly subdivided into secreted and cell membrane-tethered molecules (88, 89). Secreted mucins are further subdivided into gel-forming and non-gel-forming mucins. Examples of membrane-bound mucins include MUC1, MUC3A, MUC3B, MUC4, MUC13, and MUC16, while secreted mucins include MUC2, MUC5AC, MUC5B, MUC6, MUC7, MUC19, and MUC20.

MUC1 is the prototype membrane-tethered mucin. The MUC1 glycoprotein consists of a large extracellular ectodomain (MUC1-ED), a single-pass transmembrane domain, and an intracellular cytoplasmic domain (MUC1-CD) (90–92). The MUC1-ED is primarily composed of varying numbers (25–125) of highly O-glycosylated, 20-amino acid VNTRs, with the number of repeats being a polymorphic genetic trait. The MUC1-CD contains multiple Ser, Thr, and Tyr residues as potential phosphorylation sites. MUC1 is initially synthesized as a single polypeptide chain, but autoproteolytic cleavage at a Gly-Ser peptide bond in the endoplasmic reticulum generates two subunits that are together transported to the cell surface. The larger subunit (> 250 kDa) is derived from most of the MUC1-ED and the smaller subunit (20-30 kDa) contains a juxtamembrane region of the MUC1-ED, the membrane-spanning domain, and the MUC1-CD (93). MUC1 autoproteolysis occurs within its SEA (sea urchin sperm protein, enterokinase, agrin) domain, a 120-amino acid region highly conserved in glycosylated, mucin-like proteins (94, 95). While the two autoproteolytic cleavage fragments constitute separate polypeptide chains, they remain tightly associated on the cell surface, although they are not linked through disulfide bonds, and are only separated under extreme dissociating conditions, for example in the presence of sodium dodecyl sulfate (96). However, the MUC1-ED can be untethered from the cell surface under physiologic conditions by additional proteolytic cleavage by proteases such as neutrophil elastase (97, 98), TNF-α converting enzyme (TACE) (99), matrix metalloprotease-14 (MMP-14) (100, 101), and γ-secretase (102). Finally, an extracellular form of the MUC1-ED, MUC1-SEC, can be produced through alternative splicing of the MUC1 mRNA to introduce a translation stop codon prior to its transmembrane region (103).

The 72-amino acid MUC1-CD contains 9 Ser, 6 Thr, and 7 Tyr residues as potential phosphorylation sites. Many of these residues are located within consensus sequence binding motifs for signaling kinases and adapter proteins. Among the most well-characterized of these are phosphatidylinositol 3-kinase (PI3K), Shc, phospholipase Cγ (PLCγ), protein kinase Cδ (PKCδ), glycogen synthase kinase-3β (GSK3β), c-Src, epidermal growth factor receptor (EGFR), β-catenin, and Grb-2 (104–107). The pattern of MUC1-CD Tyr phosphorylation is similar to that of some cytokine and growth factor receptors, but unlike cytokine/growth factor receptors, the MUC1-CD is not capable of autophosphorylation.

Carbohydrate analysis of MUC1 glycans reveals a high content of sialic acid (108, 109). Because MUC1 is the major membrane-bound mucin expressed in the airways and NEU1 is the major sialidase expressed in the respiratory tract (40), studies were performed to determine the molecular relationship between NEU1 and MUC1 in airway epithelial cells. By immunohistochemical staining, airway expression of NEU1 matches with that seen for MUC1 in these same tissues, i.e. at the superficial surface of airway epithelia, including the brush border of the trachea and bronchus, but not in subepithelial tissues (40, 110, 111). Overexpression of NEU1 in cultured human airway epithelial cells increased its association with MUC1 and decreased the sialic acid content of the MUC1-ED as assessed by lectin blotting with the sialic acid-reactive lectin, *Maakia amurensis* lectin (40). In contrast, NEU1 silencing in these cells had the opposite effects. Thus, the MUC1-ED is a substrate for NEU1. However, it is unknown whether NEU1 desialylates MUCs other than MUC1, and whether NEUs other than NEU1 desialylate MUC1.

NEU1 desialylates not only MUC1, but also EGFR (40). Because MUC1 forms a molecular complex with EGFR (112–114), and NEU1 plays a role in programmed cell death (115, 116), studies were performed to examine the relationship between NEU1, MUC1, and EGFR in autophagy (117). Immunohistochemically, human triple-negative breast cancer cells expressed high levels of EGFR, but low levels of MUC1 and NEU1. The levels of two autophagy pathway proteins, PI3K and beclin-1, were positively correlated with both NEU1 and MUC1-ED levels, while only PI3K correlated with the MUC1-CD. These results led to the authors to speculate that a NEU1 – MUC1 – EGFR axis is dysregulated in breast cancer cells exhibiting reduced autophagy.

## KL-6

**Figure 2:**
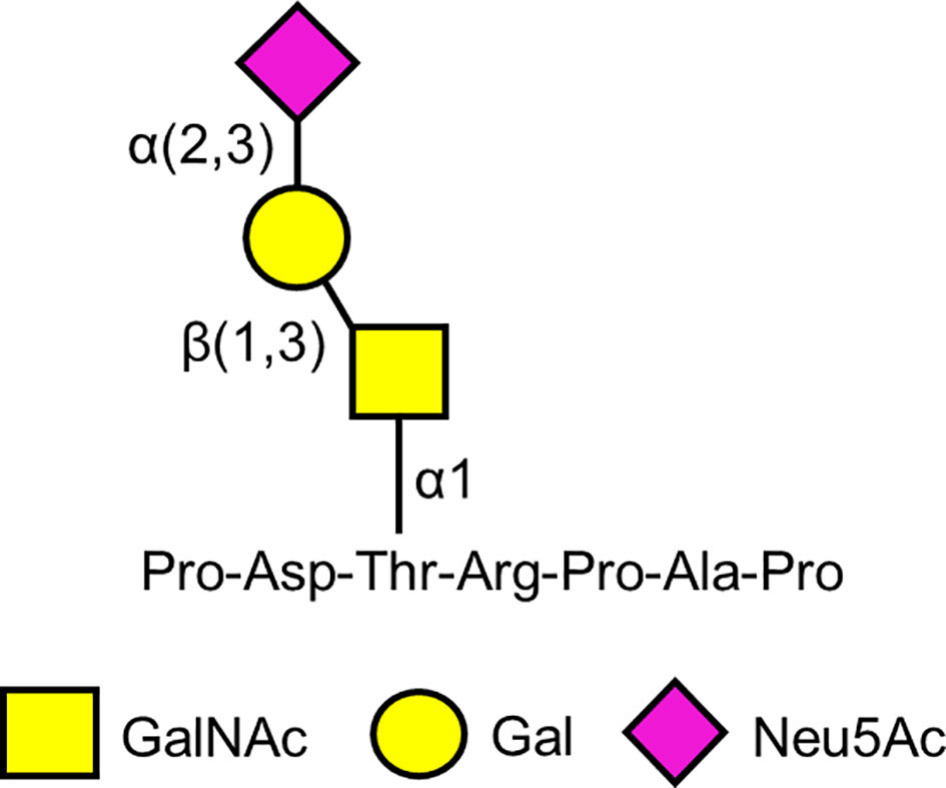
Proposed minimal antigenic structure of the KL-6 epitope based on Ohyabu et al. (120).

In 1988, a mouse monoclonal antibody, Krebs von den Lungen-6 (KL-6) (from the German “cancer from the lungs”) was described that was raised against human pulmonary adenocarcinoma cells (118). In addition to the immunizing cells, the KL-6 antibody reacted with normal human type 2 alveolar pneumocytes and bronchiolar epithelial cells, and with an unidentified high molecular weight mucin-like serum glycoprotein whose levels were increased in patients with lung adenocarcinoma. The epitope on the KL-6 antigen recognized by the KL-6 antibody was initially defined as a sialylated glycan because treatment of the purified antigen with sialidase diminished its reactivity with the cognate antibody (118). Carbohydrate analysis identified Gal, GalNAc, and Neu5Ac (sialic acid) in the KL-6 epitope (119). Subsequently, Ohyabu et al. (120) reported that the minimal antigenic structure of the KL-6 epitope consisted of the glycan, Neu5Acα2,3Galβ1,3GalNAcα, attached to the Thr residue in the peptide, Pro-Asp-Thr-Arg-Pro-Ala-Pro (**Figure 2**). This carbohydrate structure also is referred to as the 2,3-sialyl T antigen of the core 1-type O-glycan. More recent analysis suggests that the KL-6 antibody also recognizes a 6′-sulfo-Gal/GalNAc glycan epitope (121). Interestingly, the KL-6 epitope only is expressed in humans and apes and not other mammals (122).

On the basis of flow cytometric and immunohistochemical studies, KL-6 was initially categorized as a Cluster 9/MUC1 antigen in the lung tumor and differentiation antigen classification system (123). Biochemical analysis of purified KL-6 established its identity with MUC1 (119). Amino acid analysis indicated a relatively high content of Pro (19.1 mol %), Thr (13.7%), and Ser (12.3%), while carbohydrate analysis revealed enrichment of Neu5Ac (30.2% per weight), Gal (18.9%), and GalNAc (11.9%). Most convincingly, in Western blot analysis the purified KL-6 glycoprotein reacted with the established MUC1 antibody, DF3. Because the KL-6 antibody reacts with the surface of type II pneumocytes and bronchiolar epithelial cells, and KL-6 is present in bronchoalveolar lavage fluid (BALF) and serum, the mechanism through which KL-6 is transported between the cell – BALF – blood compartments has been speculated (124, 125). While it is unclear how KL-6 is released from the cell surface into the alveolar fluid that is collected as BALF, the identification of two established MUC1-ED sheddases, MMP-14 and γ-secretase, in airway epithelial cells (51) suggests a proteolytic mechanism of KL-6 shedding.

## NEUs and MUCs in Pulmonary Fibrosis

Exaggerated, often irreversible, and sometimes relentless scarring, known as fibrosis, can affect any organ or tissue. Excessive activation of fibroblasts, manifesting as their transdifferentiation into collagen-depositing, apoptosis-resistant, myofibroblasts underlies fibrosis. The ensuing replacement of the functional parenchyma with the scar tissue can become functionally and cosmetically debilitating in the skin or outright deadly when fibrosis develops in the lung, heart, kidney, or liver. There is no cure for fibrosis, and existing therapies do not consistently prevent fibrosis progression. The mechanistic involvement of NEUs in fibrosis has been suggested relatively recently, mostly in the lungs, where NEU activation promotes fibrosis (48, 52, 126–130); but also heart, with reports suggesting a profibrotic role for NEU1 (131) and an antifibrotic role for NEU3 (132). Mucins have also been suggested as mechanistic contributors to pulmonary fibrosis and, furthermore, NEUs and MUCs interplay mechanistically, as reviewed in detail below. Therefore, the following discussion focuses mostly on pulmonary fibrosis. The mechanisms of fibrosis are highly diverse, complex, redundant, and organ-specific, yet there are numerous unifying features across organ- and tissue-specific fibroses. In relevance to pulmonary fibrosis, these mechanisms include, but are not limited to, activation of transforming growth factor-β (TGF-β) (133, 134), changes in the levels of other cytokines regulating both inflammation and fibrosis (135–142), oxidative stress (143, 144), coagulation disturbances (145, 146), changes in biomechanical forces (147, 148), cellular senescence (149–155), defective autophagy (156, 157), and dysregulated epithelial cell – fibroblast crosstalk (158–161).

Pulmonary fibrosis is usually considered in the context of interstitial lung disease (ILD), an umbrella entity that comprises a large and diverse group of human disorders characterized by variously proportioned pulmonary inflammation and scarring. Idiopathic pulmonary fibrosis (IPF) represents the most severe, debilitating, and poorly understood ILD (162, 163). Connective tissue disease related ILD, especially in patients with scleroderma and rheumatoid arthritis, also poses a serious biomedical problem (164–166). Although the roles for NEU contributions to ILD have been suggested recently, early indications were observed in the past. Lambré et al. (167) first reported sialidase activity in the BALF from patients with IPF, while BALF from healthy subjects and serum from both IPF patients and controls had no such activity. Later, NEU1 mRNA and protein levels were shown to be increased in lung epithelial and endothelial cells, and fibroblasts, of IPF patients compared with healthy controls (128). In the same study, NEU1 overexpression in cultured lung fibroblasts increased the levels of collagen types I and III and MMP-14 degradation products, while NEU1 overexpression in mice increased lung collagen and TGF-β levels and promoted lung leukostasis of CD8^+^, and to a lesser extent CD4^+^, T cells. A later report from the same group demonstrated that selective pharmacological inhibition of NEU1 using C9-butyl-amide-2-deoxy-2,3-dehydro-N-acetylneuraminic acid (C9-BA-DANA), attenuated collagen accumulation in the lungs and pulmonary lymphocytosis in the acute and chronic bleomycin models of lung fibrosis (48). Taken together, these results indicated that NEU1 plays a central role in the pathogenesis of pulmonary fibrosis and NEU1-selective inhibition may offer a potential therapeutic intervention for pulmonary fibrotic diseases. Other studies have implicated a role for NEU3 in IPF pathogenesis (52, 126, 127, 129, 130).

### NEU1 – MUC1 Axis in ILD

Kohno and colleagues (168) reported that serum levels of the KL-6 antigen positively correlated with disease activity in patients with interstitial pneumonitis, including IPF, hypersensitivity pneumonitis, and sarcoidosis. Elevated levels of KL-6 also were observed in the BALF of patients with ILD and there was a positive correlation between KL-6 serum and BALF levels (125, 169). Serum KL-6 levels were demonstrated to be proportionally higher in interstitial pneumonitis patients compared with other biomarkers of disease activity, such as type III procollagen N-terminal peptide (PIIIP) and type IV collagen 7S (7S collagen), while the latter two were more sensitive markers of alveolar pneumonia (170). In subsequent investigations, elevated serum levels of KL-6 were shown to be present in a greater proportion of patients with ILD compared with other reported disease biomarkers, including sialyl-Lewis a (sLe^a^), sialyl-Lewis x (sLe^x^), surfactant protein-A, surfactant protein-D, monocyte chemoattractant protein-1 (MCP-1), and C-C motif chemokine ligand 18 (CCL18) (171–174). In addition to its value as a diagnostic biomarker of ILD, KL-6 serum levels have been reported to be of prognostic value for predicting disease severity, acute exacerbations, and patient survival (125, 175, 176). Further, the value of KL-6 as a biomarker for ILD extends to diverse ethnic populations throughout the world, despite the fact that polymorphisms in the *MUC1* gene affect serum levels of KL-6 (177–179). Based on these collective studies, KL-6 has been approved by the Japanese Health Insurance Program as a clinical diagnostic biomarker for ILD (124). Several relevant review articles can be consulted for further information on the diagnostic and prognostic value of KL-6 in IPF/ILD (125, 180–183).

KL-6 has been implicated in the pathogenesis of IPF. Purified KL-6 increased the chemotaxis of human lung and skin fibroblasts *in vitro*, which was augmented by fibronectin (119). KL-6 also promoted lung fibroblast proliferation and migration, and inhibited apoptosis, which were synergized by TGF-β (184). In a mouse model of bleomycin-induced pulmonary fibrosis, administration of anti-KL-6 antibody following bleomycin administration reduced the number of BALF inflammatory leukocytes, diminished the content of hydroxyproline in lung tissues, reduced the expression of collagen type I, TGF-β, and KL-6, increased hepatocyte growth factor (HGF) expression, and inhibited airway epithelial cell apoptosis (185). Bleomycin-induced lung fibrosis was decreased in MUC1-deficient mice, and *in vitro* silencing of MUC1 expression diminished fibroblast proliferation (186). *In vitro* cultures of lung fibroblasts and epithelial cells treated with the antifibrotic drug, pirfenidone, had reduced TGF-β-induced MUC1-CD phosphorylation at Thr-42 and Tyr-46, and decreased nuclear translocation of the MUC1-CD/SMAD3/β-catenin complex (187). MUC1-ED levels were increased in the BALF of bleomycin-challenged mice, and the NEU1-selective inhibitor, C9-BA-DANA, dose-dependently inhibited bleomycin-induced increases in MUC1-ED levels (48). Further, MUC1-ED in BALF of bleomycin-challenged mice was desialylated, and C9-BA-DANA reduced bleomycin-provoked MUC1-ED desialylation. Transgenic mice expressing human MUC1 had increased serum KL-6 levels following bleomycin challenge (122). Serum KL-6 levels were positively correlated with BALF albumin levels indicating that increases in serum KL-6 after bleomycin administration were associated with disruption of the alveolar-capillary barrier. In an experimental model of silica-induced pulmonary fibrosis using mice expressing the human *MUC1* transgene, BALF and serum levels of MUC1 were increased compared with saline controls (188). Unexpectedly, however, MUC1 knockout mice had increased lung collagen deposition and pulmonary inflammation compared with wild type littermates. Collectively, these studies have led us to propose a NEU1 – MUC1 axis as a central component of the pathogenesis of pulmonary fibrotic diseases.

Several unanswered questions remaining concerning the roles of KL-6/MUC1 and NEU1 in the pathogenesis of IPF/ILD. 1) Do all secreted/extracellular forms of MUC1 that arise from different proteolytic cleavages (elastase, TACE, MMP-14, γ-secretase) and/or alternative mRNA splicing (MUC1/SEC) contribute to the appearance of KL-6? 2) Do genetic polymorphisms that dictate the number of MUC1-ED tandem repeats influence the development of IPF? 3) Are KL-6/MUC1 molecules in the serum and BALF of IPF patients desialylated to a similar extent? 4) Do pharmacologic inhibitors that target protease-mediated MUC1-ED shedding inhibit IPF development? 5) And ultimately, what is the molecular mechanism through which NEU1-induced, KL-6/MUC1 desialylation contributes to the profibrotic, proinflammatory phenotype that characterizes IPF?

Increased KL-6 levels in BALF of ILD patients likely reflects the presence of high glycoprotein levels on the surface of regenerating type II pneumocytes (168, 189). Upregulation of MUC1-ED sheddases in these cells during disease manifestation remains an alternative possibility, and elevated levels of MMP-14 have been described in pulmonary fibrosis (190, 191). High KL-6 levels in serum also may be causally related to increased regenerating pneumocytes and/or greater alveolar capillary permeability to proteins following disruption of the alveolar-capillary barrier (124).

### Mucins as Substrates for NEU2, NEU3, and NEU4

NEU2 mRNA and sialidase activity were detected in the acellular mucin-enriched capsule of the equine pre-implantation conceptus (192). Recombinant human NEU2, expressed in *Escherichia coli* and purified to homogeneity, exhibited sialidase activity against bovine submaxillary gland mucin (BSM) (193). Although the particular mucin expressed by the bovine submaxillary gland has yet to be fully characterized, cDNA sequence analysis shows it to be highly similar to MUC19 (194–196). A human NEU4 cDNA, expressed in monkey COS-7 cells, desialylated BSM to a greater extent compared with other NEU substrates, including 4-MU-NANA, sialyllactose, and mixed bovine gangliosides (197). By contrast, while recombinant NEU3 desialylated BSM to a greater extent than 4-MU-NANA, BSM was an inferior NEU3 substrate compared with sialyllactose and bovine gangliosides. Recombinant cDNAs of both the short and long isoforms of human NEU4 (NEU4-S and NEU4-L), expressed in COS-7 cells, also exhibited *in vitro* sialidase activity against BSM (198). NEU4-S expressed in human embryonic kidney 293T (HEK293T) cells, but not NEU1, NEU2, or NEU3, desialylated the sLe^a^ mucin-expressing antigen (79). NEU4-S also exhibited enzymatic activity against the sLe^x^ antigen, while NEU3 was moderately active and NEU1 and NEU2 were unreactive against sLe^x^. Compared with untreated controls, treatment of human THP-1 macrophages with the phytochemical, thymoquinone, stimulated an increase in endogenous NEU4 activity that exhibited sialidase activity against BSM (80, 199). Therefore, in addition to the established role of NEU1 in desialylating MUC1, as discussed previously, other NEUs are capable of desialylating other MUCs. Several such mucins—potential desialylation targets of mammalian NEUs—have been suggested or demonstrated to play a role in ILD. A specific single nucleotide polymorphic (SNP) *MUC2* gene variant was significantly associated with IPF (200), and the expression levels of MUC2 protein were decreased in patients with IPF (201). Similarly, a specific SNP variant of the *MUC5AC* gene was associated with IPF (202). However, the expression of MUC5AC was reported to be either increased (203) or decreased (201, 204) in patients with pulmonary fibrosis. In a mouse model, overexpression of MUC5AC protected against inflammation and fibrosis through several mechanisms (205). These apparent discrepant results might be explained, in part, by differences in disease etiologies, patient demographics, and/or lung sampling techniques between the various studies (201, 203, 204). The expression levels of MUC4 and MUC16 were elevated in patients with IPF, with evidence for each MUC4 and MUC16 promoting the fibrotic process in collaboration with TGF-β (206–209). Of particular prominence is a strong association of a gain-of-function *MUC5B* gene promoter variant (rs35705950) with pulmonary fibrosis (210–215). To date, the *MUC5B* rs35705950 SNP remains the strongest and most reproducible risk factor for development of IPF. It should be expected that specific details of the interplay between NEU isozymes and these MUCs might transpire in the future.

## NEUs and MUCs in Infectious Diseases

### NEU1 and MUC1 in *Pseudomonas aeruginosa* Infection

*P. aeruginosa* is a Gram-negative, motile, opportunistic pathogen responsible for a wide range of human infections (216). In the respiratory tract, *P. aeruginosa* contributes to the morbidity and mortality of cystic fibrosis, chronic obstructive pulmonary disease, and bronchiectasis. *P. aeruginosa* bacteria isolated from the airway secretions of cystic fibrosis patients are tightly bound to mucins (217–219). In *in vitro* cell adhesion assays, the MUC1-ED bound to *P. aeruginosa* through bacterial flagellin, the major structural protein of the flagellar filament (220, 221). Flagellin binding to the MUC1-ED stimulates two distinct molecular events, Tyr phosphorylation of the MUC1-CD leading to activation of the extracellular signal-regulated kinase 1/2 (ERK1/2) signal transduction pathway and NEU1-catalyzed MUC1-ED shedding from the cell surface as a soluble decoy receptor (40, 49, 51, 222). MUC1-CD diminishes the host inflammatory response to *P. aeruginosa* lung infection through a mechanism involving EGFR-catalyzed Tyr phosphorylation of the MUC1-CD and inhibition of TLR signaling (104, 105, 107, 112, 223–229). As discussed above, EGFR and TLRs are established NEU1 substrates, but it is unclear to what extent NEU1-mediated desialylation of EGFR and/or TLRs impacts *P. aeruginosa*-initiated, MUC1-CD-inhibited airway inflammation.

Overexpression of NEU1 in cultured human airway epithelial cells increased *P. aeruginosa* adhesion to MUC1-expressing airway epithelia, and promoted MUC1-ED desialylation and flagellin-stimulated, MUC1-dependent ERK1/2 phosphorylation, while NEU1 silencing had the opposite effects (40). To extend these results to a physiologically relevant context, additional studies were performed to assess whether *P. aeruginosa* flagellin, the MUC1 ligand, might regulate NEU1-mediated MUC1-ED desialylation and/or *P. aeruginosa* adhesion to airway epithelial cells (51). NEU1 overexpression in cultured human airway epithelial cells increased MUC1-dependent adhesion of flagellin-expressing *P. aeruginosa* to the cells, but not the adhesion of a flagellin-deficient *P. aeruginosa* isogenic mutant (fliC¯). Treatment of these cells with purified *P. aeruginosa* flagellin dose-dependently increased bacterial adhesion, which was abolished by NEU1 silencing. In coimmunoprecipitation assays, *P. aeruginosa* flagellin increased MUC1 association with both NEU1 and the NEU1 chaperone, PPCA, in a dose-dependent manner. Airway epithelial cells treated with *P. aeruginosa* flagellin also dose-dependently increased MUC1-ED desialylation, which was abrogated by NEU1 knockdown. Because sialic acid residues can mask protease recognition sites (230), additional studies were conducted to assess the role of NEU1 and *P. aeruginosa* flagellin in MUC1-ED shedding from the cell surface (51). NEU1 overexpression and flagellin stimulation dose-dependently increased shedding of desialylated MUC1-ED into the supernatants of cultured airway epithelial cells. Moreover, the desialylated MUC1-ED that was shed in response to flagellin stimulation decreased *P. aeruginosa* adhesion to airway epithelial cell-associated MUC1-ED. Thus, the MUC1 ligand, *P. aeruginosa* flagellin, increased MUC1 association with NEU1, leading to desialylation and shedding of the MUC1-ED from the airway epithelial cell surface, and the shed MUC1-ED competitively blocked *P. aeruginosa* adhesion to MUC1-ED residing on the cell surface.

Using an intact, physiologically relevant, mouse model of *P. aeruginosa* pneumonia, further studies were performed to assess whether *P. aeruginosa*-derived flagellin might stimulate NEU1-dependent MUC1-ED desialylation and shedding (49). Mice administered intranasally with viable bacteria or purified flagellin exhibited both dose- and time-dependent increases in shed MUC1-ED in their BALF. No increases in BALF levels of MUC1-ED were evident in mice administered with the flagellin-deficient *P. aeruginosa* fliC¯ mutant strain. *P. aeruginosa* lung infection of mice also increased NEU1-MUC1 and PPCA-MUC1 association and MUC1-ED desialylation, and inhibition of NEU1 activity *in vivo* with the NEU1-selective inhibitor, C9-BA-DANA (231), abrogated both of these effects. Shed, desialylated MUC1-ED purified from the BALF of *P. aeruginosa*-infected mice diminished both flagellin and bacterial binding to cultured airway epithelial cells, and a similar effect was observed using a nonglycosylated recombinant MUC1-ED protein expressed in *E. coli*. Co-administration of the recombinant MUC1-ED protein with *P. aeruginosa* to mice also decreased bacterial lung burden, diminished BALF levels of keratinocyte-derived chemokine (KC) and tumor necrosis factor-α (TNF-α), inhibited pulmonary leukostasis, and increased 5-day mouse survival from 0% to 75%.

**Figure 3:**
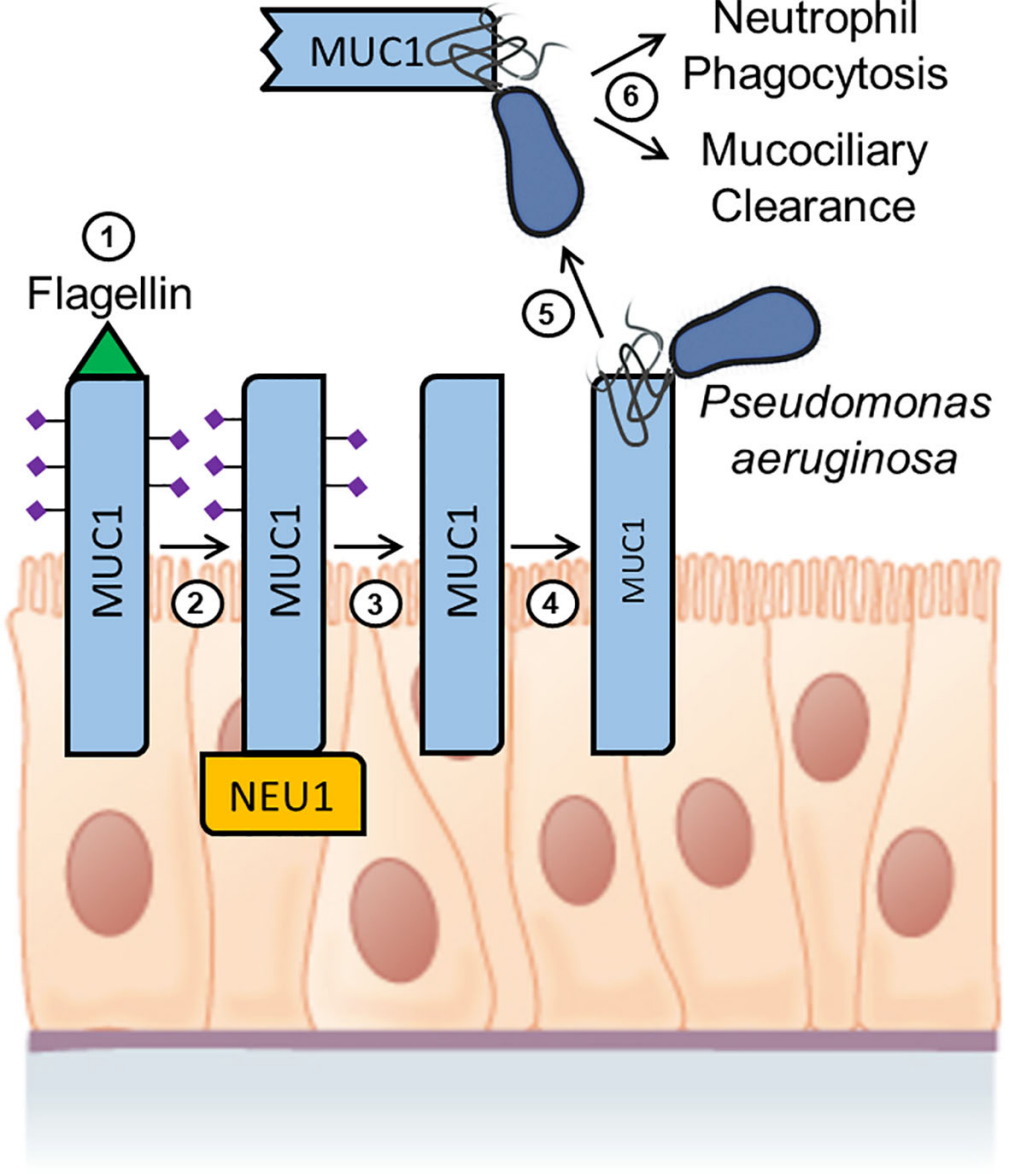
Hypothetical model for *Pseudomonas aeruginosa* flagellin-induced, NEU1-mediated MUC1-ED desialylation and shedding. Step 1. P*. aeruginosa* flagellin engages cell-associated MUC1-ED in the airway epithelium. Step 2. NEU1 is recruited to the MUC1-CD. Step 3. NEU1 desialylates the MUC1-ED. Step 4. Desialylated, cell-associated MUC1-ED binds to *P. aeruginosa* through its flagella. Step 5. The *P. aeruginosa*-MUC1-ED complex is proteolytically released from the cell surface. Step 6. The *P. aeruginosa*-MUC1-ED complex is removed from the lungs by the combined actions of neutrophil phagocytosis and mucociliary clearance. Image of the pseudostratified columnar epithelium from Anatomy and Physiology, Chapter 4: The Tissue Level of Organization, Section 4.2: Epithelial Tissue by OpenStax College, Rice University (https://openstax.org/details/books/anatomy-and-physiology) and used under Creative Commons Attribution 4.0 International (CC BY) license/Modified from the original.

To extend these results to an *in vivo* human setting, studies were conducted to determine whether shed MUC1-ED levels might be elevated in BALF of patients with *P. aeruginosa* lung infection, and whether the recombinant MUC1-ED protein might influence *in vitro* parameters of the host proinflammatory response to bacterial infection (232). BALF from patients with *P. aeruginosa* pneumonia contained higher levels of MUC1-ED, compared with either BALF from noninfected patients or patients infected with microorganisms other than *P. aeruginosa*, and shed MUC1-ED in the BALF of *P. aeruginosa*-infected patients was desialylated, presumably from the action of NEU1. *P. aeruginosa*-derived flagellin also was detected in the BALF of *P. aeruginosa*-infected patients, and a positive correlation existed between BALF levels of MUC1-ED and flagellin. Finally, *E. coli*-expressed recombinant MUC1-ED dose-dependently decreased *P. aeruginosa* motility and biofilm formation, diminished *P. aeruginosa*-stimulated interleukin-8 (IL-8) production by airway epithelial cells, and promoted neutrophil-mediated bacterial phagocytosis. Taken together, these results indicate that not only do BALF levels of shed MUC1-ED provide a diagnostic biomarker of *P. aeruginosa* lung infection, but that NEU1-mediated desialylation of the MUC1-ED generates a flagellin-targeting releasable decoy that provides a protective component of the proinflammatory response to *P. aeruginosa* at the human airway epithelial surface (**Figure 3**).

In subsequent studies, coimmunoprecipitation and glutathione-S-transferase (GST) pull down assays were performed to identify the biochemical mechanism through which NEU1 associates with MUC1 (233). Unexpectedly, NEU1 associated with the MUC1-CD, but not with the sialic acid-containing MUC1-ED. Inhibition of NEU1 enzymatic activity using C9-BA-DANA did not affect NEU1-MUC1-CD association. The NEU1 binding site was localized to the membrane-proximal 36 amino acids of the MUC1-CD, while the MUC1-CD binding site was mapped to NEU1 amino acids 1-139. NEU1-MUC1-CD association was direct and did not require the NEU1 chaperone protein, PPCA. NEU1-mediated MUC1-ED desialylation in response to *P. aeruginosa* flagellin was equivalent in cells either expressing or not expressing the MUC1-CD, indicating that NEU1-MUC1-CD interaction was not required for MUC1-ED desialylation. Finally, overexpression of NEU1 in airway epithelial cells reduced MUC1-CD association with PI3K and diminished downstream Akt phosphorylation. These results indicate that NEU1 associates with the juxtamembrane region of the MUC1-CD to inhibit PI3K-Akt signaling.

Several unanswered questions remain concerning the NEU1 – MUC1 axis. 1) What is the molecular mechanism through which engagement of *P. aeruginosa* flagellin by the MUC1-ED leads to recruitment of NEU1? 2) Do other flagellated respiratory pathogens other than *P. aeruginosa* activate the flagellin – MUC1 – NEU1 axis? 3) Can the *E. coli-*expressed recombinant MUC1-ED offer a clinically useful therapeutic intervention to treat *P. aeruginosa* lung infections either alone or in combination with anti-pseudomonal antibiotics? 4) Can measurement of MUC1-ED and/or flagellin levels in BALF be developed into a dependable and rapid diagnostic assay to identify patients with *P. aeruginosa* lung infections without the need for bacterial culture or genotyping techniques?

### NEUs and MUCs in Other Bacterial Infections

In mouse models of *E. coli*-induced sepsis or *S. enterica* serovar Typhimurium-induced colitis, or in mice administered intraperitoneally with *E. coli* lipopolysaccharide (LPS), circulating levels of both NEU1 and NEU3 were increased compared with saline controls (234). No increases in NEU1 or NEU3 were observed in TLR4 knockout mice. *Streptococcus pneumoniae*-induced murine sepsis also increased serum NEU1 and NEU3 levels, but these increases were similar in both wild type and TLR4-deficient mice, suggesting an effect of the bacterial-encoded neuraminidase, NanA. Mechanistic studies revealed that increased NEU1/NEU3 levels promoted desialylation and clearance of alkaline phosphatase by the Ashwell-Morell receptor, thereby decreasing LPS dephosphorylation and increasing TLR4-dependent host inflammatory responses. In other studies, repeated gastric administration of *S. enterica* Typhimurium or LPS increased intestinal sialidase activity and NEU3 mRNA and protein levels in the gut in a TLR4-dependent manner with a corresponding increase in alkaline phosphatase desialylation and decrease phosphatase activity (235). Oral administration of zanamivir (Relenza), an influenza virus NEU inhibitor, blocked the bacterial-induced increased intestinal NEU activity, maintained alkaline phosphatase sialylation, and prevented disease. No increases in intestinal sialidase activity, NEU expression, or alkaline phosphatase desialylation were seen following gastric administration of *S. enterica* Typhimurium to NEU3 knockout mice (236). NEU3-deficient, *S. enterica* Typhimurium-infected mice also demonstrated decreases in LPS phosphorylation, intestinal levels of proinflammatory cytokines, and gut infiltration of CD3^+^ T cells, Gr1^+^ neutrophils, and F4/80^+^ monocyte/macrophages, and protected against the development of bacterial colitis. The NEU inhibitor oseltamivir (Tamiflu), originally developed against influenza virus NEU, but also active against NEU1 (41, 237–239), reduced murine lung neutrophil infiltration, IL-17 and TNF-α levels in BALF and serum, and mortality in both experimental *E. coli* sepsis and the cecal ligation puncture model of sepsis (240).

MUC1 serves as a cell surface receptor for *Helicobacter pylori* in gastric epithelia (100, 241–246). Here, the MUC1-ED is released as a soluble decoy receptor to limit *H. pylori* infection (100, 247, 248), but the role of NEU1 in *H. pylori*-stimulated MUC1-ED shedding is unclear. MUC1 expression downregulates *H. pylori*-driven gastric inflammation (244, 249–256). MUC1 expression also influences a variety of other human bacterial infections, including those by *E. coli*, *S. enterica* Typhimurium, *S. pneumoniae*, *Staphylococcus aureus*, *Bacillus subtilis*, *Campylobacter jejuni*, and *Haemophilus influenzae* (257–267). Based on these studies and the documented role of the NEU1 – MUC1 axis in regulating airway inflammation in response to *P. aeruginosa* (40, 49, 51, 232), it is likely that this same pathway impacts the interaction of a broad array of pathogenic microorganisms with the human host. In summary, these collective studies suggest that NEUs and MUCs play important roles in regulating bacterial infections.

### NEUs and MUCs in Viral Infections

DNA methylation analysis of primary nasal epithelial cells from children with or without asthma implicated NEU1 in the host immune response to human rhinovirus-16 infection (268). Compared with non-asthmatic controls, NEU1 mRNA levels were decreased in asthma subjects with rhinovirus-16 infection, and a positive correlation between *NEU1* gene methylation and NEU1 transcript levels was observed. The hepatitis B virus core protein (HBc) increased *NEU1* gene promoter activity through NF-κB binding sites, leading to activation of ERK1/2 and NF-κB signaling pathways, and increased HBc-mediated migration and proliferation of hepatoma cells (269). Knockdown of NEU2 expression in monkey Vero or human HEp-2 cells increased hepatitis A virus replication by up to 200-fold, however the mechanism was not elucidated (270). Overexpression of mouse NEU3 in COS-7 cells diminished Newcastle Disease Virus (NDV) infection and propagation through cell-cell fusion (271). Because reduced cell surface levels of GD1a also were decreased upon NEU3 overexpression, this ganglioside was suggested to constitute an NDV receptor on host cells. The expression of NEU1, but not NEU3 or NEU4, was increased in upper and lower respiratory tract cells, and in lung infiltrating neutrophils, of SARS-CoV-2-infected patients (240). Interestingly, two recent reports have documented increased MUC1 protein levels in the airways of SARS-Cov-2-infected patients compared with healthy controls (272, 273). MUC1 also has been shown to play a critical role in several other virus infections, including those by respiratory syncytial virus, adenovirus, influenza virus, and human immunodeficiency virus (274–280). Future studies are needed to elucidate the role of NEUs, if any, in regulating the MUC1-dependent responses to virus infections. In conclusion, these investigations reveal that a variety of human and animal virus infections are impacted by the expression and activity of NEU1, NEU2, NEU3, and MUC1.

### NEU1 in Parasite Infections

*In vitro* infection of mouse J774.A1 macrophage cells with the obligate intracellular protozoan parasite, *Leishmania donovani*, reduced cell surface expression of NEU1 compared with uninfected controls (55). Parasite infection enhanced TLR4 sialylation and reduced TLR4-NEU1 and TLR4-MyD88 association. NEU1 overexpression in *L. donovani*-infected murine macrophages increased TLR4 desialylation, TLR4-NEU1 and TLR4-MyD88 association, activation of the JNK, ERK1/2, p38, and NF-κB pathways, and expression of IL-12, interferon-γ (IFN-γ), and inducible nitric oxide synthase (NOS) proinflammatory mediators, while decreasing expression of IL-4, IL-10, and TGF-β and reducing parasite burden, all compared with mock-transfected cells. In other studies, NEU1 overexpression in *L. donovani*-infected mouse macrophages reduced TLR4 ubiquitination and TLR4-siglec-E and siglec-E-SHP1 association, but increased IL-1β, IL-6, and TNF-α levels compared with nontransfected cells (281).

## NEUs in Innate and Adaptive Immunity

### NEUs and TLRs

Toll-like receptors (TLRs) are cell surface sialoglycoproteins that recognize broadly-distributed pathogen-encoded molecules to initiate MyD88- and TRIF-dependent intracellular signaling pathways that activate host innate immune responses (282). It is now established that sialidases, principally NEU1, regulate TLR-dependent innate immunity (**Table 2**).

**Table 2:** NEU-associated cells, signaling pathways, and cytokine responses for selected immune/inflammatory processes.

Immune/Inflammatory Process	NEU Isozyme	Cells	Signaling Pathways	Cytokine Responses	References
TLR2, TLR3 activation	NEU1	Macrophages	NF-κB	ND	(53)
TLR4 activation	NEU1	Macrophages	NF-κB	↑ IL-6, TNF-α	(53, 57)
TLR4 activation	NEU1	Adipocytes	NF-κB	↑ IL-6, MCP-1	(54)
TLR4 activation	NEU1	Kidney mesangial cells	ERK, p38	↑ IL-6, GM-CSF, MIP-1α	(283)
TLR4 activation	NEU1	HEK293T cells	NF-κB	ND	(58)
TLR4 activation	NEU1	Microglia	ND	↑ IL-6, MCP-1	(56)
TLR4 activation	NEU3	Dendritic cells	ND	↑ IL-6, IL-12, TNF-α	(66)
TLR4 activation	NEU4	Macrophages	NF-κB	↑ IL-1β, IL-6, TNF-α, IFN-γ	(80, 199)
TLR7, TLR9 activation	NEU1	Macrophages	NF-κB	↑ TNF-α, MCP-1	(44)
PMA activation	NEU1	Macrophages	ND	↑ IL-1β, IL-6, TNF-α	(64)
*Staph. aureus*, *E. coli* infection	NEU1	Macrophages	FcγR, Syk kinase	ND	(31)
*Leishmania donovani* infection	NEU1	Macrophages	JNK, ERK, p38, NF-κB	↑ IFN-γ, IL-12 ↓ IL-4, IL-10, TGF-β	(55)
*Salmonella enterica* Typhimurium infection	NEU3	T cells, Neutrophils, Macrophages	ND	↑ IL-1β, TNF-α, IFN-γ, IL-10, TGF-β	(236)
*Pseudomonas aeruginosa* infection	NEU1	Airway epithelial cells	PI3K, Akt	ND	(233)
Hepatitis B virus infection	NEU1	Hepatoma cells	ERK, NF-κB	ND	(269)
Concanavalin A activation	NEU1	T cells	ND	ND	(284, 285)
Anti-CD3 antibody activation	NEU1	CD4^+^ T cells	ND	↑ IL-2, IL-4	(286)
Anti-CD3/CD28 antibody activation	NEU1, NEU3	CD4^+^ T cells	ND	ND	(287)
Anti-CD3/CD28 antibody activation	NEU1	CD4^+^ T cells	ND	↑ IL-2, IL-4, IL-13, IFN-γ	(288)
Anti-CD3/CD28 antibody activation	NEU3	CD4^+^ T cells	ND	↑ IL-2, IL-13, IFN-γ	(288)
Anti-CD3/CD28 antibody activation	NEU1	T cells	ND	↑ IFN-γ	(65)
*Dermatophagoides farinae* activation	NEU1	CD4^+^ T cells	ND	↑ IL-4, IL-5, IL-13	(30)
Ovalbumin activation	NEU3	FoxP3^+^ T cells	ND	ND	(289)
Obesity-induced insulin resistance	NEU1	FoxP3^+^, Th17^+^ T cells	ND	ND	(290)
Systemic lupus erythematosus	NEU1	Kidney mesangial cells	ND	↑ IL-6, MCP-1	(291)
Rheumatoid arthritis	NEU2, NEU3	CD19^+^ B cells, CD138^+^ plasma cells	ND	ND	(292)
Type 1 diabetes	NEU1	Liver, Muscle cells	Insulin receptor kinase, Akt	ND	(39)
Type 1 diabetes	NEU1	Liver cells	p38, Akt	ND	(38)
Type 1 diabetes	NEU1	Hepatoma cells	Insulin receptor, Insulin receptor substrate-1	ND	(293)
Type 1 diabetes	NEU3	Adipocytes, Muscle cells	Insulin receptor, Insulin receptor substrate-1, Grb-2	ND	(294)
Type 1 diabetes	NEU3	Liver cells	Insulin receptor substrate-1, PPARγ	ND	(295)
Type 1 diabetes	NEU3	Adipocytes	Akt	ND	(296)
Diabetic cardiomyopathy	NEU1	Cardiomyocytes	AMPK, SIRT3	ND	(131)
Inflammatory bowel disease	NEU3	Intestinal mucosa	ND	ND	(297)
Inflammatory bowel disease	NEU1	Peripheral blood mononuclear cells	ND	ND	(298)

ND, Not determined.

TLR ligands, including LPS, poly(I:C), *Mycobacterium butyricum*, and zymosan, induced sialidase activity in mouse BMC-2 and primary bone marrow macrophages that was associated with NF-κB activation and the production of nitric oxide, IL-6, and TNF-α (53). In these same studies, NEU1, but not NEU2, NEU3, or NEU4, colocalized with TLR2, TLR3, and TLR4 on the cell surface of unstimulated mouse macrophages, which increased following stimulation with zymosan, poly(I:C), or LPS, respectively. NEU1 knockout mice had reduced granulocyte-colony stimulating factor (G-CSF), IL-1Rα, IL-6, KC, and macrophage inflammatory protein-2 (MIP-2) production in response to LPS administration compared with NEU1-expressing mice. In other studies, NEU1- and NEU4-deficient macrophages had reduced LPS-induced TLR4-MyD88 complex formation and NF-κB activation compared with wild type littermates (57, 80, 199). Following LPS stimulation of mouse dendritic cells (DCs), NEU1 translocated from the lysosome to the cell surface where it associated with and desialylated TLR4, and NEU1 silencing diminished LPS-stimulated TNF-α production (70). In these same studies, NEU1 deficiency in murine hematopoietic cells or *in vivo* treatment with the sialidase pharmacologic inhibitor, 2-deoxy-2,3-dehydro-N-glycolylneuraminic acid (Neu5Gc2en), protected mice against lethal LPS challenge. NEU1 silencing in mouse 3T3-L1 adipocytes increased TLR4 sialylation and decreased LPS-stimulated NF-κB nuclear translocation and IL-6 and monocyte chemoattractant protein-1 (MCP-1) production (54). NEU1 pharmacologic inhibition with oseltamivir decreased LPS-stimulated IL-6, granulocyte-macrophage colony-stimulating factor (GM-CSF), and MIP-1α production in mouse primary renal mesangial cells through a TLR4 → p38/ERK pathway (283). Treatment of mouse RAW264.7 macrophages with LPS decreased NEU1 levels on the cell surface, and NEU1 silencing promoted LPS-induced tolerance, while NEU1 overexpression had the opposite effect (73). Finally, treatment of RAW264.7 cells with TLR7 or TLR9 agonists promoted TLR7-NEU1 and TLR9-NEU1 association, NF-κB activation, and TNF-α and MCP-1 production, all of which were reversed by NEU1 silencing or pharmacologic inhibition with oseltamivir (44).

In human studies, *NEU4* was identified as the strongest locus associated with TLR4 stimulation in a genetic analysis comparing LPS-treated primary monocytes with untreated cells (299). Overexpression of NEU1, but not NEU3, in HEK293T cells dose-dependently increased LPS-stimulated, TLR4-mediated NF-κB activation and treatment with the broad spectrum sialidase inhibitor, 2-deoxy-2,3-dehydro-N-acetylneuraminic acid (Neu5Ac2en or 2-deoxy-NANA) reduced NF-κB activation (58). Mechanistically, it was proposed that NEU1-mediated desialylation of TLR4 unmasked TLR dimerization sites to promote receptor activation and initiation of the host proinflammatory response (53, 57). Additional studies revealed that NEU1, MMP-9, and G protein-coupled receptors (GPCRs) formed a multiprotein complex on the cell surface wherein GPCR agonists activated GPCR signaling to stimulate MMP-9-dependent NEU1 activation through removal of the elastin-binding protein, thereby allowing for TLR desialylation and initiation of host inflammation (44, 45, 47). Further investigations revealed NEU1 – MMP-9 cross-talk in combination with the GPCR agonist, neuromedin B, as necessary for EGFR activation and downstream intracellular signaling (41). LPS activation of mouse microglial BV-2 cells and rat primary microglia increased cell surface sialidase activity and desialylation of the cell surface (300, 301), which was abolished by NEU1 knockdown (56). NEU1 knockdown in microglia also reduced LPS-stimulated IL-6 and MCP-1 expression, while NEU1 overexpression had the opposite effects (56). Using a cell surface biotinylation assay, NEU1 and TLR4 were localized in close proximity (within 100 nm) on the cell surface of LPS-activated BV-2 cells, and LPS treatment of the cells increased TLR4 desialylation. Collectively, studies in both human and mouse experimental systems indicate that NEU1, and to a lesser extent NEU4, regulate TLR-driven intracellular signaling and host innate immunity.

### NEUs and PMNs

Polymorphonuclear leukocytes (PMNs) are blood-borne leukocytes that play a critical role in the host innate immune response to infectious pathogens through microbial phagocytosis, release of soluble anti-microbial compounds, and generation of neutrophil extracellular traps (NETs) (302). Sialidase activity was identified in the primary and secondary granules of human PMNs as well as on the plasma membrane, and PMN activation induced translocation of the enzymatically-active sialidase from the granules to the cell surface (303). In addition, PMN homotypic aggregation and adhesion to nylon or plastic surfaces was inhibited by 2-deoxy-NANA. In other studies, a rabbit antibody prepared against the *Clostridium perfringens* NEU cross-reacted with a sialidase expressed on the surface of IL-8-stimulated human PMNs, but not on unstimulated cells, and with sialidase proteins in PMN lysates and granule preparations (304). This same antibody inhibited human PMN sialidase activity *in vitro* and diminished both pulmonary leukostasis in mice administered with cobra venom factor and intrapulmonary transendothelial migration of PMNs into the bronchoalveolar compartment of IL8-administered mice. In subsequent experiments, the sialidase antibody was shown to recognize recombinant human NEU3, but not NEU1, and inhibited NEU3 sialidase activity against the synthetic fluorogenic substrate, 4-MU-NANA (305). In other studies, NEU1, NEU2, and NEU3 mRNAs were detected in unstimulated murine PMNs and IL-1β stimulation of human PMNs increased NEU1 and NEU2, but not NEU3, expression (288). LPS-stimulated human PMNs had decreased amounts of sialic acid on their surface that was inhibited by oseltamivir and zanamivir (240). Both NEU1 inhibitors also reduced PMN-mediated phagocytosis and killing of *E. coli*, LPS-driven PMN activation, phorbol 12-myristate 13-acetate (PMA)-stimulated reactive oxygen species production, and LPS-mediated NET formation.

Utilizing the NEU3 cross-reactive antibody, Sakarya *et al.* (306) reported reduced, antibody-dependent PMN adhesion to pulmonary vascular endothelial cell monolayers *in vitro*, which was associated with PMN activation. Preincubation of endothelial cell monolayers with activated PMNs rendered the cells hyperadhesive for freshly added PMNs *via* PMN sialidase-dependent autodesialylation. Desialylation of both the CD11b and CD18 protein subunits of β2-integrin, through mobilization of an endogenous PMN sialidase, unmasked activation epitopes on both proteins and increased binding to intracellular adhesion molecule-1 (ICAM-1) (21). NEU1 was expressed on the surface of PMNs and colocalized with CD18, the latter being increased following PMN activation. Desialylation of immobilized ICAM-1 increased leukocyte arrest *in vivo* and treatment with 2-deoxy-NANA diminished LPS-stimulated leukocyte adhesion to ICAM-1. Taken together, these results indicate that NEU1, NEU2, and NEU3 regulate the activities of PMNs.

### NEUs and Monocytes, Macrophages, and Dendritic Cells

Macrophages isolated from NEU1 knockout mice exhibited reduced phagocytosis of Gram positive (*S. aureus*) and Gram negative (*E. coli*) bacteria and phosphorylation of FcγR and Syk kinase, and treatment of cultured NEU1-deficient macrophages with purified mouse NEU1 restored their phagocytic activity (31). NEU1-knockout macrophages displayed increased lysosomal exocytosis as a consequence of reduced NEU1-mediated desialylation of lysosomal-associated membrane protein-1 (LAMP-1) (34). High levels of NEU1 mRNA were detected in unstimulated human monocytes, while NEU2 and NEU3 were expressed at much lower levels (288). *In vitro* stimulation of human monocytes with GM-CSF to promote differentiation into macrophages temporally increased sialidase activity for the 4-MU-NANA substrate up to 14-fold after 7 days in culture (307). Correspondingly, NEU1 and NEU3, but not NEU4, mRNAs and proteins were increased following monocyte-to-macrophage differentiation. Following PMA-stimulated differentiation of human primary monocytes and the THP-1 monocyte cell line to macrophages, NEU1, but not NEU3, protein levels increased and NEU1 translocated from the lysosomes to the plasma membrane (64). NEU1 silencing reduced *E. coli* phagocytosis by PMA-induced macrophages and diminished their production of IL-1β, IL-6, and TNF-α. Differentiation of human monocytes into DCs following *in vitro* culture in the presence of GM-CSF and IL-4 increased sialidase activity and elevated the mRNA and protein levels of NEU1 and NEU3, but not NEU4 (66). Further, monocyte-to-DC differentiation in the presence of zanamivir or 2-deoxy-NANA to inhibit NEU1 and NEU3 activities decreased LPS-stimulated IL-6, IL-12, and TNF-α production. Overexpression of NEU1 in THP-1 macrophages increased IL-1β and TNF-α production while NEU1 silencing had the opposite effect (308). In these same studies, NEU1 silencing in human primary M1 macrophages suppressed IL-1β and TNF-α production. PMA-induced differentiation of THP-1 cells to M0 macrophages increased intracellular sialidase activity and diminished sialoglycoconjugate levels on the cell surface (308). However, further polarization of M0 macrophages into M1 or M2 macrophages did not affect the amount of sialic acid on the cell surface.

NEU1 expression was increased in human circulating monocytes isolated from patients with myocardial infarction compared with healthy controls, and high NEU1 levels were observed in macrophages isolated from atherosclerotic plaques (308). In an animal model of atherosclerosis, NEU1-deficient mice exhibited reduced monocyte and lymphocyte infiltration into atherosclerotic lesions compared with NEU1-sufficient mice (309). Reductions in serum and liver nonhigh-density lipoprotein (nonHDL) cholesterol levels, atherosclerotic lesion size, and lesion macrophage numbers were observed in apolipoprotein E (ApoE) knockout mice expressing hypomorphic levels of NEU1 (NEU^hypo^) compared with ApoE^-/-^ littermates (310). NEU1^hypo^ ApoE knockout mice also had reduced circulating PMNs, but increased numbers of M2 macrophages, compared with ApoE knockout mice expressing normal NEU1 levels. Finally, treatment of ApoE^-/-^ mice with the general sialidase inhibitor, 2-deoxy-NANA, reduced liver nonHDL cholesterol levels and atherosclerotic lesion size, compared with saline controls. In another study, recombinant human NEU1 and NEU3 desialylated apolipoprotein B 100 in human low-density lipoprotein (LDL) *in vitro*, and ApoE-NEU1 and ApoE-NEU3 double knockout mice, or ApoE knockout mice treated with pharmacologic inhibitors of NEU1 or NEU3, exhibited reduced atherosclerosis compared with ApoE single knockout mice or ApoE null mice treated with vehicle control (18). In a mouse model of ischemia/reperfusion (I/R) cardiac injury, elevated sialidase activity, and NEU1, PPCA, and β-galactosidase mRNA and protein levels, were seen in the heart compared with sham controls (311). These authors also reported that NEU^hypo^ mice subjected to I/R injury had diminished proinflammatory and increased anti-inflammatory macrophage numbers compared with controls. Further, cardiomyocyte-specific NEU1 overexpression *in vivo* was associated with cardiac hypertrophy, but no changes in cardiac inflammation, following I/R injury compared with wild type littermates. Binding of pro-atherogenic elastin-derived peptides (EDP) to the elastin receptor complex (ERC), a heterotrimeric association of NEU1, PPCA, and the elastin binding protein, a splice variant of β-galactosidase (312), led to NEU1-mediated desialylation of the CD36 scavenger receptor and augmented uptake of oxidized LDL by human macrophages *in vitro* (22). In a subsequent report, EDP binding to the ERC also promoted NEU1-β2-integrin association and NEU1-driven β2-integrin desialylation in human monocytes, as well as NEU1-ICAM-1 association and NEU1-mediated ICAM-1 desialylation in human umbilical vein endothelial cells (23). As a result, EDP binding to the ERC promoted monocyte adhesion to and migration across endothelial cell monolayers through NEU1. In conclusion, these studies strongly suggest that NEU1 regulates monocyte, macrophage, and DC differentiation and function (**Table 2**).

### NEUs and Lymphocytes

Landolfi and coworkers (284) reported that mouse T lymphocytes possess an endogenous sialidase activity that increased following *in vitro* stimulation with the T cell mitogen, concanavalin A, and sialidase activity was controlled by the *NEU1* locus in the murine major histocompatibility complex. Mouse strains carrying the H-2^v^ haplotype (SM/J, B10.SM) exhibited reduced sialidase activity compared with other haplotypes. Previously, the SM/J mouse strain had been reported to possess deficient sialidase activity (313). Subsequent analysis revealed that a point mutation in the *NEU1* gene, resulting in a Leu-to-Ile substitution, was responsible for the deficient sialidase activity in the SM/J stain (314). Increased sialidase activity following T cell activation was associated with desialylation of cell surface glycoproteins (285). Mouse T cell activation through a mixed lymphocyte reaction (MLR) with allogeneic B cells also resulted in increased sialidase activity and desialylation of T cell surface glycoproteins (315). Wipfler *et al.* (316) reported that total sialidase activity was greatest in unstimulated human peripheral blood lymphocytes (PBLs), intermediate in thymic T cells, and lowest in tonsillar T and B cells. At the mRNA level, NEU1 was expressed at approximately 2.5-fold greater levels in PBLs compared with thymocytes and tonsillar T and B cells, while NEU3 was expressed at up to 125-fold greater levels in thymic T cells compared with PBLs and tonsillar T and B cells. In a different study, NEU1 and NEU3 transcripts were identified in murine thymic T cells (20). Crude membrane fractions of mouse thymocytes had sialidase activity that was inhibited by 2-deoxy-NANA and the NEU1-specific inhibitor, C9-BA-DANA. CD5 was identified as a substrate for thymocyte cell surface NEU1 by peanut agglutinin (PNA) lectin blotting and anti-CD5 antibody immunoprecipitation studies. A subpopulation of murine thymic lymphocytes was identified that co-expressed surface IgG, β2-integrin, and NEU1 (Neu-medullocytes) (317, 318). Real-time PCR studies revealed that NEU2 was the major sialidase expressed in Neu-medullocytes with NEU1 detected at low levels (319). Of five NEU2 mRNAs arising as a result of alternative mRNA splicing (variants A, B, C, D, N), only variant B was identified in murine thymocytes (320). Up to 40% of NEU2 variant B sialidase activity was found in the membrane fraction of transfected COS-7 cells. An analysis of mouse thymic lysates prepared with different detergents suggested that NEU2 was a peripheral membrane protein, rather than a transmembrane or GPI‐anchored protein, with enzymatic activity at pH 7.0 but not pH 4.5 (78). It should be noted that other studies have established NEU2 as a cytosolic enzyme with a pH optimum of 6.0-6.5 and an amino acid sequence lacking protein domains typical of transmembrane or GPI-anchored proteins (12, 84, 85).

The levels of IL-2 and a T helper 2 (Th2) cytokine, IL-4, were increased following *in vitro* anti-CD3 antibody stimulation of murine spleen cells, and reduced levels of IL-4, but not IL-2 or IFN-γ, were seen upon anti-CD3 antibody activation of SM/J splenocytes (286). Addition of exogenous IL-4 during CD4^+^ T cell activation enhanced NEU1 sialidase activity and cell surface levels of asialoGM1 ganglioside, while activation of T cells in the presence of 2-deoxy-NANA diminished IL-4 production. In other studies, treatment of IL-4-primed CD4^+^ T cells with the GM3 ganglioside diminished IL-4, IL-5, and IL-13 production, but had no effect on IL-2 or IFN-γ, through a mechanism involving mobilization of intracellular calcium (321). Further, anti-CD3 antibody stimulation of NEU1-deficient B10.SM strain CD4^+^ T cells reduced intracellular calcium levels compared with unstimulated cells. A COOH-terminal tetrapeptide, Tyr-Gly-Thr-Leu, was suggested to constitute an internalization signal in the NEU1 protein that targets it to the lysosome in human lymphocytes, fibroblasts, and COS-7 cells (62). Upon Tyr phosphorylation of the tetrapeptide in activated lymphocytes, NEU1 sialidase activity was unchanged, but the sialidase was redistributed from the lysosome to the cell surface. NEU1 and NEU3 enzyme activities and mRNA levels were increased in human CD4^+^ T cells activated with anti-CD3 and anti-CD28 antibodies (288). NEU3 was the major sialidase expressed in the activated T cells. Overexpression of NEU1 in human Jurkat T cells increased IL-2, IL-4, IL-13, and IFN-γ expression following CD3/CD28 costimulation, while these same cytokines, with the exception of IL-4, were produced in NEU3-overexpressing cells. CD3/CD28 costimulation of human primary T cells increased IFN-γ production, which was inhibited by zanamivir and 2-deoxy-NANA (65). CD3/CD28 costimulation also increased NEU1 mRNA levels and sialidase activity with minimal increase in NEU3 activity, heightened NEU1 and PPCA cell surface expression, and increased the levels of desialylated cell surface glycoconjugates, all compared with unstimulated cells. While these studies suggested that NEU1, and possibly NEU3, might be linked to Th2 immunity, they also highlight the apparent contradiction to the studies reviewed above implicating NEU1 in the Th1 response (65, 66, 308).

Altered expression of surface sialoglycoconjugates is a characteristic feature of apoptosis (287). Treatment of Jurkat T cells with the proapoptotic drug, etoposide, increased sialidase activity and desialylated cell surface glycoconjugates during the early phase of apoptosis, which was reversed by 2-deoxy-NANA (322). In subsequent studies, etoposide-induced apoptosis was associated with increased cell surface GM3, reduced GD3, and increased NEU3, but not NEU1, mRNA levels (323). Changes in GM3, GD3, and NEU3 levels were blocked by caspase inhibition. Phytohemagglutinin/IL-2-induced human T cell blasts undergoing UV- or staurosporine-induced apoptosis exhibited decreased surface staining for terminal α2,3- and α2,6-linked sialic acids (324). Desialylation of surface glycoconjugates also was observed on microparticles derived from apoptotic lymphoblast membranes. Plasma membrane-derived apoptotic microparticles had increased sialidase activity on their surface (325). Treatment of viable Jurkat cell lysates with caspase-3 increased sialidase activity to the same degree as that of apoptotic cell lysates, consistent with a predicted caspase-3 proteolytic cleavage site at Asp-135 in the NEU1 protein. Further, the caspase inhibitor, zVAD, decreased sialidase activity in etoposide-induced apoptotic Jurkat cells. Not only was cell surface NEU1 responsible for desialylating Jurkat T cell surface glycoconjugates in a *cis*-acting manner, but also in *trans* on the surface of co-incubated human erythrocytes (67).

In a mite allergen model of allergic asthma, *in vitro* exposure of *Dermatophagoides farinae* (Derf)-sensitized CD4^+^ T cells to the antigen increased desialylation of cell surface glycoconjugates, augmented binding of hyaluronic acid (HA) to its cognate receptor, CD44, and heightened NEU1, but not NEU2 or NEU3, transcript levels (30). In NEU1-deficient cells from SM/J mice, CD44 receptor activity for HA was reduced, as were reductions in BALF eosinophil and Th2 cell numbers, Th2 cytokine levels, and airway hyperresponsiveness following Derf challenge, compared with cells expressing normal NEU1 levels. Subsequent studies using *in vitro*-differentiated ovalbumin-specific CD4^+^ T cells revealed that NEU1 and NEU3 mRNA levels were greater in Th2, compared with Th1, cells (326). Collectively, these studies indicated that NEU1 is required for HA-CD44 interaction and development of acute asthmatic inflammation, and suggested that NEU1-mediated desialylation of CD44 on CD4^+^ T cells alters the ability of CD44 to interact with HA (28). In subsequent studies, analysis of mouse CD4^+^ T cell subsets revealed that while NEU1 mRNA was expressed at approximately equal amounts in Th1, Th2, Th17, and induced regulatory T (iTreg) cells, NEU3 transcript levels were greater in iTreg cells compared with the other cell subsets (289). CD4^+^ activated Treg (FoxP3^+^CD62L^−^) spleen cells expressed higher levels of NEU3 transcripts compared with resting Treg (FoxP3^+^CD62L^+^) and FoxP3^−^ cells, and overexpression of NEU3 in naïve CD4^+^ T cells upregulated FoxP3 expression. In a mouse model of obesity-induced insulin resistance, the percentages of CD4^+^FoxP3^+^ Treg cells and NEU1 levels were decreased, while the percentages of CD4^+^Th17^+^ cells and the levels of the NEU1-targeting microRNA, miR-23b-3p, were increased, all compared with nonobese controls (290). Administration of the anti-obesity phytochemical, acacetin, reversed these effects, while administration miR-23b-3p exacerbated the outcomes. Co-administration of acacetin and miR-23b-3p restored the obesity-related downregulated levels of NEU1, while NEU1 overexpression offset the effects of miR-23b-3p on the Treg/Th17 cell ratio. The authors concluded that acacetin reverses insulin resistance in obese model mice by regulating the Treg/Th17 cell balance through a miR-23b-3p – NEU1 axis. Finally, in human studies, differential gene expression analysis identified 7 genes related to the lysosomal pathway, including NEU1, that were downregulated in the airways of asthma patients compared with healthy controls, suggesting that lymphocyte lysosomal dysfunction might be involved in the pathogenesis of allergic asthma (327).

Vitamin D is a potent immunomodulator (328).Vitamin D-binding protein (DBP), also referred to as the group-specific component (Gc) protein in humans, is the main transporter of vitamin D in the circulation. Part of the immune-modulating effects of vitamin D are mediated through the conversion of Gc/DBP to macrophage activating factor (MAF) (329). In humans, the Gc protein contains a mucin-type O-linked trisaccharide composed of Thr-linked GalNAc with dibranched sialic acid and Gal termini, while MAF contains only the Thr-linked monosaccharide GalNAc (330). A two-step sequential action of β-galactosidase on the surface of B cells followed by surface-expressed NEU1 on T cells was identified as being responsible for the conversion of Gc to MAF (331–333). In mice, DBP is not sialylated and only the action of β-galactosidase on the Gal-GalNAc disaccharide was required to deglycosylate DBP to MAF. Taken together, these results indicate that NEU1, and to a lesser extent NEU3, play in important role in regulation of adaptive immunity through their effects on T and B lymphocytes (**Table 2**).

## NEUs and MUCs in Autoimmune Diseases

Autoimmune diseases comprise a diverse collection of pathological conditions originating from a dysregulated immune response to a self tissue or organ. As discussed above, NEUs play a critical role in the activity of Treg cells (289, 290) suggesting the possible involvement of NEUs in the pathogenesis of autoimmunity. Further, MUC1 has been suggested as a checkpoint inhibitor on T cells (334), implicating MUC1, and possibly other membrane-bound mucins, in the development of autoimmune diseases. This section reviews the role of NEUs and MUCs in the context of four selected autoimmune diseases, systemic lupus erythematosus (SLE), rheumatoid arthritis (RA), type 1 diabetes (T1D), and inflammatory bowel disease (IBD).

### Systemic Lupus Erythematosus

SLE is an autoimmune disease typically characterized by the presence of anti-nuclear antibodies (ANA) and anti-extractable nuclear antigen (anti-ENA) antibodies with immune complex deposition in multiple organs, particularly the kidneys (335). A growing body of evidence suggests a role for NEU1 in the pathogenesis of lupus nephritis (283, 336–338). More specifically, NEU1 expression and catalytic activity were increased in the kidneys of MRL/lpr lupus mice (339), and kidney mesangial cells from NEU1-haplodeficent mice had reduced cytokine expression and secretion in response to LPS or lupus serum (LS) compared with NEU1-expressing mice (340). In other studies, overexpression of NEU1 in cultured mouse MES13 kidney mesangial cells dose-dependently increased spontaneous IL-6 and MCP-1 production and treatment of primary mesangial cells from MRL/lpr lupus mice with heat-aggregated IgG (HA-IgG), a mimic of immune complex deposition, increased NEU1 expression (291). Moreover, pharmacologic inhibition of NEU1 activity with oseltamivir reduced HA-IgG- or LS-stimulated IL-6 and MCP-1 production. In human studies, proteomic analysis of renal tissues comparing patients with proliferative lupus nephritis *vs*. healthy controls revealed NEU1 as the greatest up-regulated protein of 48 identified proteins (341). NEU1 immunohistochemical staining and urinary excretion were significantly elevated in lupus nephritis patients compared with controls. Finally, flow cytometric analysis of human B cells from SLE patients revealed a positive correlation between the cell surface ratio of sialyltransferase ST3Gal-1 and NEU3 with disease severity (342).

SLE patients with serositis had greater MUC16 levels and disease duration compared with SLE patients without serositis (343). Pseudo–pseudo Meigs’ syndrome is a rare manifestation in SLE patients presenting with ascites, pleural effusion, and elevated serum MUC16 levels unrelated to malignancy (344–346). MUC20 transcript levels were increased with the progression of lupus in the kidneys of MRL/lpr lupus-prone mice (347). Interestingly, mouse NEU1 (348) and mouse and human MUC20 (347) are highly expressed in kidneys compared with other organs, but it is unknown whether MUC20 might be a NEU1 substrate.

### Rheumatoid Arthritis

RA is an autoimmune disease of unknown etiology characterized by synovitis and bone erosion (349). A positive correlation between B cell surface ST3Gal-1/NEU3 ratio and RA disease severity was seen in patients with mild or high disease activity, but not patients in non-remission (342). In a follow-up study, B cell surface NEU3 levels were found to positively correlate with RA disease activity (350). NEU1 catalytic activity was increased in the blood and liver of rats with Freund’s adjuvant-induced RA (351). In a mouse model of bovine collagen type II-induced RA, pharmacologic inhibition of NEU2 and NEU3 activity with zanamivir dose-dependently diminished CD19^+^ B cells and CD138^+^/TACI^+^ plasma cells numbers, anti-collagen type II autoantibody levels, disease activity, and total sialidase activity in arthritic joints (292). Corresponding, increased sialylation of circulating IgG and α2,3-linked surface sialylation of CD138^+^/TACI^+^ plasma cells was noted.

MUC1 protein levels were elevated on T cells isolated from the joint fluid of a patient with RA (352). The combination of lung ultrasound and serum MUC1 levels, in addition to current markers, was proposed for screening and following patients with ILD associated with RA (353). A greater number of RA patients expressed MUC3 protein in synovial lining cells and synovial macrophages compared with normal controls (354). The *MUC5B* gene promoter variant rs5705950 was linked to an increased susceptibility to pulmonary fibrosis in RA patients (213, 215, 355). Increased salivary MUC7 sulfation was detected in RA patients compared with healthy controls (356). Synovial fluid cells from RA patients contain a distinct population of MUC18-positive T cells (357), and these cells were identified as a biomarker for synovial membrane angiogenesis in RA patients (358).

### Type 1 Diabetes

T1D is an autoreactive disease arising from immune destruction of insulin-producing pancreatic β cells (359). Both NEU1 and NEU3 have been implicated in the pathogenesis of T1D. NEU1 transcript levels and catalytic activity were diminished in two strains of diabetes-prone mice compared with nondiabetic littermates (360). Treatment of cultured rat L6 myoblasts with purified mouse NEU1 promoted desialylation of both the insulin receptor (IR) and insulin-like growth factor-1 receptor (IGF-1R) compared with untreated controls (36). Mice deficient in NEU1 activity and fed a high fat diet (HFD) developed glucose intolerance and insulin resistance compared with mice expressing normal levels of NEU1, and HEK293 cells overexpressing both NEU1 and the insulin receptor kinase (IRK) exhibited increased IRK-NEU1 association and NEU1-mediated IRK desialylation (39). Liver and muscle cells from NEU1-deficient, HFD-fed mice had decreased IR phosphorylation and downstream Akt activation, and treatment of HEK293 cells overexpressing IR and NEU1 promoted IR-NEU1 association and NEU1-mediated IR desialylation. Administration of EDPs to normal mice expressing the NEU1-containing ERC increased IR-NEU1 association and NEU1-mediated IR desialylation in muscle tissues, both of which were reversed by 2-deoxy-NANA (37). Murine hepatocytes treated with EDPs in the context of nonalcoholic steatohepatitis linked to insulin resistance had decreased hepatic growth factor receptor (HGFR) phosphorylation as a consequence of NEU1-mediated HGFR desialylation (43). Overexpression of NEU1 in HEK293 cells increased IR desialylation and dimerization, and NEU1 overexpression in human HepG2 liver cells reversed palmitate-induced insulin resistance (38). In these same studies, pharmacologic upregulation of NEU1 expression and activity with ambroxol in mice led to desialylation of the IR, increased IR Tyr phosphorylation and Akt signaling, and improved glucose tolerance and insulin resistance in mice exposed to a HFD. NEU1 expression was increased in the spleen, liver, and kidneys of streptozotocin (STZ)-treated diabetic rats (361). Similarly, NEU1 protein levels were increased in cardiomyocytes in the STZ mouse model of diabetic cardiomyopathy, and NEU1 silencing protected against STZ-induced cardiomyopathy and myocardial fibrosis, inflammation, and apoptosis through an AMPK-SIRT3 pathway (131). Inhibition of NEU1 activity with anti-NEU1 antibody or oseltamivir reduced insulin-stimulated IGF-1R and insulin receptor substrate-1 (IRS-1) phosphorylation in human fibroblasts, and promoted IR-NEU1 association in rat HTC hepatoma cells (69). Further, pharmacologic inhibition of MMP-9 in HTC cells dose-dependently reduced insulin-stimulated NEU1 sialidase activity, and treatment of NEU1-deficient human fibroblasts with olanzapine, an antipsychotic drug associated with insulin resistance, increased NEU3 sialidase activity, both compared with vehicle controls. Finally, a variety of different G protein-coupled receptor agonists dose-dependently increased NEU1 sialidase activity, IR activation, and IR and IRS-1 phosphorylation in human IR-overexpressing HTC cells (293).

NEU3-overexpressing mouse 3T3-L1 adipocytes and L6 myocytes, and muscle tissue from human *NEU3* transgenic mice, had decreased insulin-stimulated IR signaling, and administration of insulin to *NEU3* transgenic mice induced NEU3 Tyr phosphorylation and NEU3-Grb-2 association (294). *In vivo* overexpression of NEU3 in the livers of insulin-sensitive or insulin-resistant mice increased hepatic levels of glycogen, triglycerides, peroxisome proliferator-activated receptor γ (PPARγ), ganglioside GM1, and IRS-1 phosphorylation, compared with control mice, suggesting that NEU3 regulates hepatic insulin sensitivity and glucose tolerance (295). Obese strain rats fed a HFD had reduced NEU3 expression in adipose tissue through an intracellular palmitate-driven, Akt-dependent pathway (296). Glucose-stimulated insulin release by rat INS-1D pancreatic β cells was increased by 2-deoxy-NANA, and blood insulin levels were greater in NEU3-deficient mice compared with wild type controls (362). The relationship between insulin resistance and intestinal hypoxia was explained, in part, by the activation of hypoxia-inducible factor-2α (HIF-2α) in hypoxic, HFD-fed mice leading to NEU3 activation (363).

MUC1 downregulation is necessary for human and mouse fetal-uterine implantation, and diabetic mice expressed increased MUC1 mRNA and protein levels at fetal-uterine implantation sites compared with nondiabetic controls (364). STZ-induced diabetic mice administered with sodium hyaluronate eye drops for treatment of diabetic ocular surface disease exhibited increased MUC5AC expression in the conjunctival epithelium compared with saline controls (365). STZ-induced diabetic rats had decreased MUC10 protein levels in salivary submandibular glands compared with nondiabetic controls (366). Transcriptome analysis of nucleolar protein p120 (NOP2)-deficient *vs*. NOP2-expressing human colon cancer cells identified the *MUC19* gene as significantly upregulated, and T1D as a gene pathway most significantly affected, by NOP2 silencing (367). Future studies are needed to determine whether NEUs regulate MUC-associated diabetes.

### Inflammatory Bowel Disease

IBD comprises a collection of idiopathic, intestinal autoimmune diseases, the most common being Crohn’s disease (CD) and ulcerative colitis (UC) (368). CD typically affects the small and large intestines, while UC primarily involves the colon and rectum. Elevated expression of intestinal NEU3 (297) was correlated with diminished levels of intestinal alkaline phosphatase (IAP) in patients with colitis (369), and human genetic deficiency of IAP is associated with IBD (370), suggesting that NEU3 may play a role in the pathogenesis of IBD. Compared with controls, a 2.0-fold increase in GM3 ganglioside levels and an 8.3-fold increase in NEU3 protein levels were reported in the intestine of IBD patients (297). Because NEU3 is responsible for the conversion of disialylated GD3 to monosialylated GM3, the authors speculated that increasing dietary GD3 intake may be beneficial for treatment of IBD patients. A SNP, rs4947331, encoding a minor T allele located in the 3’ untranslated region of the *NEU1* gene was significantly associated with the presence of CD in North Indian and Dutch populations (298). It was suggested that the NEU1 pharmacologic inhibitor, oseltamivir, might be applicable to treat CD patients. Serum sialic acid levels were increased, presumably as a consequence of increased NEU catalytic activity, and positively correlated with established markers of disease activity, in CD patients (371, 372). Meta-analysis of six genome-wide association studies in CD patients identified the *MUC1* gene as a candidate for disease susceptibility (373). Elevated expression of multiple mucin gene products, including MUC1, MUC4, and MUC13, were reported in IBD patients compared with controls (374–376). By contrast, another study reported decreased expression of these MUCs in IBD patients (377). MUC1 knockout mice had more severe forms of Th1- and Th2-induced colitis and increased Th17 cell-mediated responses in the colon, compared with MUC1-expressing mice (378). Studies in both human and animal model systems indicated that MUC1 regulates the progression of IBD to colon cancer (379–384). Alterations in MUC2 intestinal expression and glycosylation were reported in IBD patients (385, 386). MUC2 knockout mice had greater intestinal inflammation and reduced body weight gain compared with MUC2-expressing mice, despite equal food intake, and exhibited intestinal microbiome, short chain fatty acid, and inflammatory cytokine profiles similar to that of IBD patients (387, 388). The Winnie mouse strain possessing a *MUC2* gene point mutation encoding a Cys-to-Tyr missense mutation develops spontaneous colitis with many of the same pathologic features seen in UC patients (389). Other MUCs associated with CD and UC disease include MUC3, MUC5AC, MUC5B, MUC6, MUC16, MUC19, and MUC20 (390–394). Additional studies are needed to establish a causal link between the expression and catalytic activity of NEUs with MUCs in IBD. In summary, the development and progression of SLE, RA, T1D, and IBD are influenced by both NEUs (**Table 2**) and MUCs.

## Discussion

In this review, we have attempted to summarize historical and recent research on the roles of mammalian NEUs and MUCs, both individually and together, in the context of fibrotic and immune-mediated human diseases. First, general features of the 20-member MUC family and 4-member NEU family were summarized, including an updated tabulation of selected known and putative NEU1 glycoprotein substrates relevant to fibrotic and inflammatory processes. Particular attention was paid to the KL-6 glycoprotein, a soluble form of the membrane-bound MUC1-ED. The concept of a NEU1 – MUC1 axis in IPF/ILD was presented. Evidence supporting the NEU1 – MUC1 axis included publications demonstrating that NEU1 regulates pulmonary collagen deposition, lymphocytosis, and fibrosis in human and mouse models of IPF (128), KL-6 serves as a diagnostic and prognostic biomarker for, and mediator of, IPF/ILD (125, 180–183), and most importantly, NEU1 pharmacologic inhibition reverses bleomycin-induced increases in MUC1-ED desialylation and shedding from the cell surface (48). Finally, the influence of NEU1 on human infections by bacterial, viral, and parasitic pathogens, and the impact of NEUs and MUCs on four selected human autoimmune diseases was discussed.

A limitation of our analysis was that the expanse and complexity of this field prevented us from an in-depth exploration of the broader NEU/MUC biology. We utilized a reductionist approach, which on the one hand, allowed for a focused analysis, but on the other hand, limited the scope by excluding related topics. Only mammalian NEUs and MUCs were considered. Bacterial or viral exosialidases, endosialidases, or trans-sialidases were not discussed despite their direct relevance to disease processes. We did not consider catalytic mechanisms of NEU activity but instead emphasized the functional consequences relevant to health and disease. Further reducing the complexity and sharpening the focus of this review, only the immune-, inflammation-, and fibrosis-related functions of mammalian NEUs and MUCs were discussed; the effects on other processes, e.g., atherosclerosis, diabetes, or cancer, were mentioned as far as they related to the immune, inflammatory, or connective tissue aspects of those processes. Moreover, the complexity of the mechanisms controlling NEU1 elevation or decline in the diseases were not considered. We only reviewed the functional consequences of glycoprotein desialylation, with substantially less attention devoted to desialylation of another major group of NEU substrates, glycolipids, and its functional sequelae. We did not reflect on the balancing action of sialyltransferases, which covalently attach sialic acid to glycans in contrast to NEUs, which remove it. The biological effects of free sialic acid were not considered either. An important topic that was not discussed in depth relates to recent developments in discovery and characterization of mammalian NEU isozyme-specific inhibitors; these have been considered here only to the extent that helps in reviewing NEU function. We refer the reader interested in those aspects of NEUs excluded here to numerous review articles that illuminate these topics (12, 15, 17, 81, 395). Even with such reductions, the information summarized in this review is rather voluminous and complicated, yet we hope that it is sufficiently focused thematically to be useful.

In summary, human NEUs and MUCs, particularly NEU1 and MUC1, contribute alone, and in symphony, to the development and progression of a variety of fibrotic, inflammatory, and fibro-inflammatory human pathologies. Future studies focusing on the details of the NEU1 – MUC1 relationship are expected provide new insights into the mechanisms of selected disease processes characterized by dysregulated fibrosis and inflammation that may someday lead to novel bedside therapeutic interventions for the benefit of humankind.

## Data Availability Statement

The original contributions presented in the study are included in the article/supplementary material. Further inquiries can be directed to the corresponding author.

## Author Contributions

All authors conceived and designed the review outline, contributed to the writing and critical review of the manuscript, and approved the final version of the manuscript.

## Funding

This work was supported by grants from the National Institutes of Allergy and Infectious Diseases (AI-144497) and the U.S. Department of Defense (W81XWH1910056) to EL and the U.S. Department of Defense (PR202031) to IL.

## Conflict of Interest

The authors declare that the research was conducted in the absence of any commercial or financial relationships that could be construed as a potential conflict of interest.

## Publisher’s Note

All claims expressed in this article are solely those of the authors and do not necessarily represent those of their affiliated organizations, or those of the publisher, the editors and the reviewers. Any product that may be evaluated in this article, or claim that may be made by its manufacturer, is not guaranteed or endorsed by the publisher.
